# The Role of Vitamin D in Diabetic Nephropathy: A Translational Approach

**DOI:** 10.3390/ijms23020807

**Published:** 2022-01-12

**Authors:** Charlotte Delrue, Reinhart Speeckaert, Joris R. Delanghe, Marijn M. Speeckaert

**Affiliations:** 1 Department of Nephrology, Ghent University Hospital, 9000 Ghent, Belgium; Charlotte.Delrue@ugent.be; 2 Department of Dermatology, Ghent University Hospital, 9000 Ghent, Belgium; Reinhart.Speeckaert@ugent.be; 3 Department of Diagnostic Sciences, Ghent University, 9000 Ghent, Belgium; Joris.Delanghe@ugent.be; 4 Research Foundation-Flanders (FWO), 1000 Brussels, Belgium

**Keywords:** diabetes mellitus, diabetic nephropathy, vitamin D

## Abstract

According to several animal and human studies, vitamin D appears to play a significant role in the development of diabetic nephropathy. However, the possible renoprotective effect of vitamin D and its influence on the reversal of already existing renal damage remains doubtful. At this moment, there are a few hypotheses concerning the underlying molecular and genetic mechanisms including the link between vitamin D and inflammation, oxidative stress, and extracellular matrix accumulation. The present review aims to investigate the potential role of vitamin D in the development of diabetic kidney disease from a translational approach.

## 1. Introduction

Diabetes mellitus (DM), one of the most common chronic diseases [1], is the leading cause of end-stage renal disease (ESRD) in the Western world [2,3,4]. This metabolic disorder is characterized by hyperglycemia due to inadequate production of insulin (type 1 DM (T1DM)) or insulin resistance (type 2 DM (T2DM)). The classic symptoms of hyperglycemia are polyuria, polydipsia, weight loss, occasionally polyphagia, and blurred vision. In most cases, the disease develops progressively, and the classic symptoms can remain unnoticed by the patient in the early stages of the disease [5]. Being one of the major microvascular complications of DM [6,7], diabetic kidney disease (DKD) or diabetic nephropathy (DN) is observed in approximately 20–40% of diabetic patients [8].

Vitamin D deficiency is worldwide an increasing medical problem [9,10]. At this moment, there is no consensus on the definition of vitamin D deficiency or the optimal concentration of total serum 25-hydroxyvitamin D (25(OH)D), which is the sum of 25(OH)D_2_ and 25(OH)D_3_. Most researchers and available guidelines (e.g., the Endocrine Society clinical practice guideline) consider vitamin D deficiency in the general population as a 25(OH)D_3_ level of less than 20 ng/mL (50 nmol/L) [11]. However, other organizations have slightly different definitions and recommend maintaining levels above 30 ng/mL (75 nmol/L) in categories at risk [12,13]. 

The most critical factor that confounds efforts to develop consensus clinical and nutritional public health guidelines for interpreting serum 25(OH)D concentrations is the substantial variability in many assays that have been used over the years to measure 25(OH)D [14]. The fat-soluble prohormone vitamin D plays an important role in calcium and bone metabolism, cell proliferation and differentiation, and immunoregulation [15]. The human body has two main sources of vitamin D: diet (20%) and exposure of the skin to sunlight (80%) [10,16]. 

Vitamin D is metabolized to 25(OH)D_3_ in the liver, followed by another hydroxylation in the kidneys that results in 1,25-dihydroxyvitamin D [1,25(OH)_2_D_3_] [9]. 1,25(OH)_2_D_3_ reflects the reserve status of vitamin D [17]. The production of 1,25(OH)_2_D_3_ in the kidneys is regulated by the plasma parathyroid hormone and serum calcium and phosphor concentrations [10]. Vitamin D is transported through the human body in the bloodstream by binding to vitamin D binding protein (VDBP). 

VDBP is a low-molecular-weight protein of 58 kDa, which predicts the bioavailability of 25(OH)D_3_ in the bloodstream. The complex formation of VDBP/25(OH)D_3_, its filtration, and the reabsorption of this complex in the proximal renal tubular cells are critical for the retrieval and activation of vitamin D. People with renal damage, like diabetic patients with DKD, have increased urinary VDBP concentrations [18]. 

The effects of vitamin D are mediated through binding to its receptor (VDR), which is present in a variety of tissues in the human body, including the kidneys [1,2], and more specifically in the proximal and distal tubular epithelial cells, in the glomerular parietal epithelium, in the collecting duct cells, in the mesangial cells, and in the podocytes as well as in the juxtaglomerular apparatus [3,4]. 

This indicates that the kidneys play a crucial role in vitamin D metabolism by controlling the reabsorption of calcium and phosphate and by regulating the synthesis of the active form of vitamin D [5]. The binding of vitamin D to the VDR activates the dimerization of the retinoid X receptor (RXR). The heterodimer binds to vitamin D responsive elements (VDRE) in the DNA sequence of genes regulated by this active metabolite, causing a conformational change in the VDR with the recruitment of cofactors. 

These cofactors will bind to specific DNA locations, leading to a modification in the expression of its target genes, transcriptional response, and protein formation [1,6,7]. The *VDR* gene is located on the long arm of chromosome 12 (12q13.11), containing 14 exons [8,9]. Currently, the association of *VDR* gene polymorphisms with DM and its microvascular complications has become a hot topic for intensive research [10]. 

The aim of the present review was to investigate the role of vitamin D in the development of DKD with a focus on the possible underlying molecular and genetic mechanisms.

## 2. Animal Studies

Several animal studies have shown that 25(OH)D_3_ concentrations are significantly lower in DN compared to healthy controls, suggesting that vitamin D plays a significant role in the development of DN [15,16]. In mice and rats with DN, a significantly lower expression of the *VDR* gene has been observed [18]. More specifically, investigation of VDR protein expression in the kidney tissue of rats showed a mainly nuclear VDR expression in glomerular podocytes and cytoplasmatic expression in tubular epithelial cells of healthy kidneys, whereas a significantly reduced expression of VDR was found in streptozotocin (STZ)-induced diabetic rats [18]. 

Studies in rats and mice have investigated whether supplementation with vitamin D might have a renoprotective role in the process of developing DN [15,16,18,19,20,21,22]. In the study of Hamzawy et al. [20], 30 diabetic rats were divided into three groups: group 1 (the control group), group 2 (the DN group), and group 3 (the vitamin D-treated DN group). The vitamin D-treated DN group showed a significant reduction (*p* < 0.05) in serum creatinine and urinary albumin excretion rate (UAER) after 8 weeks compared to the DN group. Similar results were observed in another study [15]. 

In STZ-treated DBA/2J mice, treatment with low-dose vitamin D, in the group with human transgenic VDR, almost entirely blocked the onset of DN [22]. In another DN study with rats [23], combined vitamin D and insulin treatment resulted in a significantly lower UAER in comparison with the individual vitamin D and insulin treatment groups (*p* < 0.05). Vitamin D supplements might reverse the histopathological changes caused by DN [15,16,18,20,21,22,23]. DN rats treated with calcitriol (1,25(OH)_2_D_3_) showed remarkable histopathologic changes compared to DN rats without treatment (*p* < 0.05) [10,20,24]. 

In addition, elevated albuminuria was lowered by calcitriol treatment in DN rats (*p* < 0.05). Light microscopy showed that silencing of the *VDR* gene eliminated the renoprotective effect of vitamin D therapy [10,20,22]. As aforementioned above, VDR seems to play a significant role in the pathophysiology of DN [10,19,20]. In STZ-induced diabetic rats, the DN group treated with a daily calcitriol supplement showed recovery of VDR expression in diabetic kidneys [18]. In an STZ-induced diabetic model in VDR knockout (VDR-KO) mice, VDR-KO mice developed more severe albuminuria than STZ-induced diabetic wild-type mice [16]. Injection with human transgenic VDR in STZ-treated DBA/2J mice resulted in a significant decrease in albuminuria compared to the wild-type controls [22].

## 3. Human Studies

### 3.1. Vitamin D and Diabetes Mellitus

Multiple studies have demonstrated that the prevalence of 25(OH)D_3_ deficiency was significantly higher in diabetic groups (both T1DM and T2DM) compared to healthy controls [25,26,27,28,29,30,31,32,33]. Type 1 diabetic patients had lower 25(OH)D_3_ concentrations compared to healthy controls [30], a phenomenon that was also observed in type 2 diabetics [32]. Complementary to these results, a significant negative correlation was observed between 25(OH)D_3_ concentrations and HbA1c (r = −0.277, *p* < 0.0001) [34]. However, in contrast to the previous findings, a retrospective study with 557 patients showed no significant lower serum 25(OH)D_3_ levels in those with T2DM compared to healthy controls [35].

Several authors have published an association between lower levels of vitamin D and a higher risk of DM [1,25,31,36,37,38,39,40]. In the prospective Nurses’ Health Study, which followed 83,779 women, who had no history of DM, cardiovascular disease, or cancer at baseline, regarding the development of T2DM, they found that vitamin D and calcium intake were inversely associated with the risk of development of T2DM [39]. Complementary to these results, a case-control study (*n* = 2224) showed a significantly higher risk for development of T2DM in patients with a 25(OH)D_3_-deficiency (≤20 ng/mL) (OR: 2.53; 95% CI: 1.81–3.45; *p* < 0.001) [31]. 

### 3.2. Vitamin D Deficiency and Diabetic Nephropathy 

The association between vitamin D deficiency and microvascular complications in diabetic patients is significantly higher than that in non-diabetics [25,29,30,32,34,41,42,43,44,45]. There is an increased risk for DN in patients with a vitamin D deficiency [28,35,40,44,46,47,48,49,50,51]. A cohort study of 1193 participants in the Diabetes Control and Complications Trial (DCCT) showed that type 1 diabetics with a 25(OH)D_3_ concentration less than 20 ng/mL had a 65% higher risk for the development of microalbuminuria (95% CI: 1.07–2.54; *p* = 0.03) compared to patients with a 25(OH)D_3_ concentration at a minimum of 30 ng/mL [40]. 

In a randomized controlled trial (RCT), type 2 diabetic patients treated with renin-angiotensin-system (RAS) blockers and with a 25(OH)D_3_ concentration (<15 ng/mL) had a faster decline in eGFR in comparison to patients with a 25(OH)D_3_ level of >15 ng/mL [48]. Another study demonstrated a significant decrease in 1,25(OH)_2_D_3_ concentration in patients with an eGFR decrease from 60 mL/min/1.73 m^2^ to <15 mL/min/1.73 m^2^ (*p* < 0.05), but no significant changes were observed when the eGFR decreased from >90 mL/min/1.73 m^2^ to 60 mL/min/1.73 m^2^ [52]. No significant changes in 25(OH)D_3_ concentration were observed with worsening renal function. 

Several studies have indicated that 25(OH)D_3_ concentrations seem to be significantly lower in DN patients, indicating that vitamin D deficiency is more prevalent in DN patients [25,30,33,34,35,42,49,50,51,52,53,54]. In a retrospective observational study (*n* = 300), patients with T2DM and CKD were 1.7-times more likely to have a vitamin D deficiency when compared to the diabetics without CKD [42]. A cross-sectional study with 479 T2DM patient showed a significantly negative correlation between 25(OH)D_3_ concentrations and urinary albumin:creatinine ratio (UACR) (r = −0.315, *p* < 0.0001) [34]. 

There was a strong positive significant correlation between 25(OH)D_3_ concentrations and eGFR (r = 2.785, *p* < 0.001). In T2DM patients at different stages of DN (*n* = 502), significantly lower serum concentrations of 25(OH)D_3_ were reported in microalbuminuric (UAER 30–300 μg/mg) (*n* = 171) and macroalbuminuric subjects (UAER > 300 μg/mg) (*n* = 130) vs. normoalbuminuric patients (UAER < 30 μg/mg) (*n* = 201) (*p* < 0.01) [29]. A significantly lower value of 25(OH)D_3_ was found in the macroalbuminuric vs. the microalbuminuric group (*p* < 0.01). 

Complementary to these results, different studies proved that the prevalence of DN was high in T1DM and T2DM patients with low 25(OH)D_3_ concentrations [29,31,43,46,54,55]. In contrast, another small study with T2DN patients (*n* = 63) showed no significant correlation between the baseline serum concentration of vitamin D metabolites and UACR [56]. No significant difference in vitamin D concentration was detected in T1DM children with DN (*n* = 18) compared to the patients without nephropathy (*n* = 40) [57]. 

In a study investigating the potential to diagnose T2DN based on 25(OH)D_3_ concentration, ROC analysis showed that the optimal cut-off concentration was 10.5 ng/mL with a sensitivity of 82.6% and a specificity of 72.7% (AUC at 0.807; 95% CI: 0.764–0.849) [34]. Some studies showed that urinary levels of VDBP (UVDBP) were higher in DN patients and could be early markers for DN [58,59,60,61]. The UVDBP concentrations were significantly (*p* < 0.001) higher in diabetic patients with DN compared to patients without DN [58]. A positive and significant correlation (r = 0.823, *p* < 0.001) with the 24-h urinary protein excretion was also demonstrated. UVDBP was significantly different among patients with normoalbuminuria, microalbuminuria, macroalbuminuria, and the control group (*p* < 0.001) [59].

### 3.3. Vitamin D Treatment and Diabetic Nephropathy

In addition to the higher risk of DN in patients with vitamin D deficiency, multiple interventional studies have shown a recovery of renal function after vitamin D therapy [27,56,62,63,64,65]. An RCT was performed in 48 T1DN patients to examine the effects of a 12-week paricalcitol treatment (starting dose was 1 µg daily if plasma parathyroid hormone levels were <53 pmol/L (<500 pg/mL), or, if higher, the starting dose was 2 µg paricalcitol daily) on renal function [62]. 

This study showed a significant reduction in the UAER and eGFR during paricalcitol therapy compared to placebo (*p* = 0.03 and *p* < 0.001, respectively). A prospective observational study (*n* = 63) examined whether an oral cholecalciferol treatment over a 4-month period (40,000 IU weekly in patients with vitamin D deficiency defined as ≤16 ng/mL (40 nmol/L), and 40,000 IU monthly in patients with vitamin D insufficiency defined as 16 ng/mL (40 nmol/L) to 32 ng/mL (80 nmol/L)) decreased the albuminuria in T2DN patients [56]. 

A significant reduction in UACR was seen at both time points (months 2 and 4) compared to the baseline UACR (*p* = 0.0011 at 2 months, *p* = 0.0201 at 4 months). A prospective randomized trial investigated the effect of calcium supplementation with or without calcitriol supplements in type 2 diabetics with CKD stage 2–4 and hypovitaminosis D (*n* = 50) [26]. The diabetic group treated with calcium supplementation (calcium carbonate 500 mg daily) only showed a significant elevation in serum creatinine (*p* = 0.03), while serum creatinine remained stable in the group treated with both calcium and calcitriol supplementation (calcium carbonate 500 mg + calcitriol 0.5 µg daily). 

Although several well-designed observational and interventional studies have demonstrated a causal relationship between vitamin D and the risk of the development of DN, the results are still controversial and there are still a few interventional trials that have shown no statistically significant effect of vitamin D supplementation [25,53,66,67,68]. A double-blind RCT (*n* = 51) showed that treatment with oral vitamin D supplements (50,000 IU weekly) for three months resulted in no statistically significant difference in UACR in patients with proven T2DN and vitamin D deficiency [66]. 

In a cross-sectional study with 119 T2DM patients, treatment with calcitriol (0.5 µg daily for 2 months) in those with a vitamin D deficiency/insufficiency resulted in a reduction in albuminuria, though the difference was not significant [53]. A double-blind RCT evaluated the effect of vitamin D supplements on oxidative/anti-oxidative markers in vitamin D deficient T2DN patients (*n* = 50) [68]. 

A group of 25 patients was treated with 1,25-dihydroxycholecalciferol (50,000 IU/week) for 8 weeks, while another group of 25 subjects received a placebo. After completing the vitamin D treatment, no significant reduction in an oxidative or significant increase in anti-oxidative parameters could be detected. In addition, no significant changes were observed in eGFR and serum creatinine between intervention and placebo groups. However, there was a significant reduction in proteinuria (*p* < 0.0001) in the vitamin D-treated group. 

## 4. Molecular Mechanisms behind the Potential Renoprotective Effect of Vitamin D in Diabetic Nephropathy 

Multiple studies have investigated the possible underlying mechanism of the renoprotective effect of vitamin D in DN. Inflammation seems to play an important role in the development of DN, which has been investigated in animal DKD studies by lipopolysaccharide (LPS) induction [69]. LPS is the ligand to TLR4, which induces the release of inflammatory factors, including interleukin (IL)-6, IL-10, IL-15, and IL-18. Some of these inflammatory factors, e.g., IL-6 and IL-15, activate the Janus kinase (JAK)-signal transducer and activator of transcription 5 (STAT5)-signaling pathway via phosphorylation of STAT (p-STAT). 

The activated p-STAT binds to nuclear DNA of the VDR to regulate the transcription of VDR. High dose of IL-15 itself can cause a massive pro-inflammatory reaction of IL-1, IL-6, and tumor necrosis factor-alpha (TNF-α) by LPS-activated macrophages [33]. In diabetic patients with renal damage, there seems to be a downregulation of VDR expression [33,70]. In comparison to healthy controls, treatment with LPS plus IL-15 resulted in a significant decrease of VDR expression in monocytes (*p* < 0.05) in DM2 patients with or without DN. 

In addition, the VDR expression was considerably lower in the DN group compared to non-DN diabetic patients (*p* < 0.05), and induced a massive pro-inflammatory response. In the LPS plus IL-15 treated monocytes, there was a significantly higher expression of nuclear p-STAT5 and co-expression of VDR-p-STAT5 complexes in DM2 and DN uremic patients, compared to healthy controls (*p* = 0.016). This upregulation was more significant in DKD patients compared to diabetic controls [33]. 

The potential anti-inflammatory and immunomodulatory effects of 1,25(OH)_2_D_3_ via VDR and STAT5 crosstalk were also evaluated in human monocytes incubated with sera from DM2 patients and DN patients with uremia [33]. After pretreatment with 1,25(OH)_2_D_3_, monocytic VDR mRNA and protein expression on nuclei and cell membrane was significantly up-regulated in T2DM and DN uremia groups. In addition, p-STAT5 expression decreased significantly compared to LPS plus IL-15 treatment alone (*p* < 0.05), and p-STAT5 expression levels did not change significantly compared to healthy controls [33]. 

**Figure 1:**
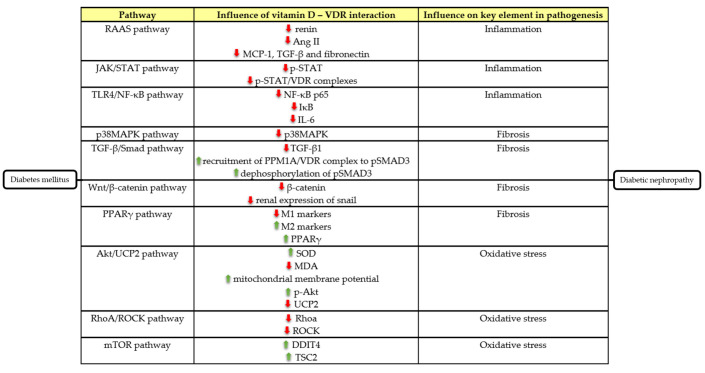
Underlying molecular mechanisms explaining the potential renoprotective capacity of vitamin D in diabetic nephropathy.

Treatment with 1,25(OH)_2_D_3_ stimulates the formation of vitamin D/VDR complexes and the exhibition of VDR DNA-binding sites. These binding sites can be attached by p-STAT5 resulting in p-STAT5/VDR complexes, which induce the formation of anti-inflammatory cytokines and inhibit the secretion of pro-inflammatory cytokines [24]. These findings suggest that the anti-inflammatory effects of vitamin D therapy might be conducted via the JAK/STAT5-signaling pathway (Figure 1) [24,33]. Monocytes exposed to LPS and IL-15 expressed significantly higher levels of IL-6 and monocyte chemoattractant protein-1 (MCP-1) in DN patients compared to healthy controls and DM2 patients (both *p* < 0.01) [33]. 

These concentrations were not influenced by treatment with 1,25(OH)_2_D_3._ Similar results were obtained in a prospective study with DM1 patients with vitamin D deficiency of insufficiency [27]. DM1 patients supplemented with calcitriol (0.25 microgram daily) for 6 months showed a significant decline in serum and urinary cytokines (MCP-1, transforming growth factor-beta (TGF-β), IL-6, and TNF-α) and proteinuria, without alleviations in hyperglycemia and β-cell functions. This implicates that the mechanism of reduction in proteinuria might be due to a reduction in inflammation rather than amelioration of the glucose metabolism [27]. 

Vitamin D may play a renoprotective role in DN by negative regulation of the renin-angiotensin-aldosterone system (RAAS) by suppressing renin expression [71]. An increased renin expression in the kidney and an increased angiotensin II concentration in plasma was detected in VDR-null mice compared with wild-type mice [72]. Following unilateral ureteral obstruction in VDR-null mice, there was an upregulation of extracellular matrix proteins and profibrogenic and proinflammatory factors such as MCP-1 and TGF-β. 

Treatment with losartan resulted in a reversal of these effects, suggesting that VDR activation attenuates renal fibrosis at least in part by suppressing the RAAS [73]. In subtotally nephrectomized rats, paricalcitol significantly reduced the mRNA concentrations and protein expression of renin, the renin receptor, and angiotensinogen [74]. Vitamin D deficiency is associated with increased circulating angiotensin II concentrations in man and blunted renal plasma flow responses to infused angiotensin II. 

These findings indicate both systemic and intrarenal RAAS activation [75]. Combined VDR activation and RAAS inhibition resulted in synergistic effects in mouse models for both type 1 [76] and type 2 diabetes mellitus [77]. More specifically, combined treatment with losartan and paricalcitol completely prevented albuminuria, and suppressed the induction of MCP-1, TGF-β, and fibronectin. 

This therapy was more effective compared with treatment with losartan or paricalcitol alone, and reversed the decline of slit diaphragm proteins, leading to the restored glomerular filtration barrier structure and markedly reduced glomerulosclerosis. Paricalcitol may be added to treatment in patients with DN who are also receiving RAAS inhibitor therapy to further reduce albuminuria [78]. 

The selective VDR activation with paricalcitol for the reduction of albuminuria in patients with type 2 diabetes (VITAL study) demonstrated the additional antiproteinuric effect of paricalcitol in diabetic patients on stable treatment of RAAS inhibition, suggesting the comparable synergistic effect in patients with DN [79]. In pre-dialysis DM2 patients, the activation of VDR might blunt albuminuria by a reduction of the urinary angiotensinogen concentrations, reflecting the intra-renal RAAS status [65].

Another hypothesis suggests that the potential anti-inflammatory effect of 1,25(OH)_2_D_3_ could occur via the toll-like receptor 4 (TLR4) and nuclear factor kappa B p65 (NF-κB p65) pathway [24]. Treatment with LPS and IL-15 resulted in significantly higher levels of TLR4 mRNA in DM2 patients with or without DN compared to healthy controls (*p* = 0.006). IL-15 and TLR4 mRNA concentrations were significantly higher in the DN group compared to DM2 patients without DN (both *p* < 0.05).

No significant differences were found for TLR9 mRNA levels. Pretreatment with 1,25(OH)_2_D_3_ did not affect the mRNA concentrations of IL-15, TLR4, or TLR9. After treatment with LPS and IL-15, there was a significant increase in protein levels of NF-κB p65 and a significant decrease in inhibitor of NF-κB (IκB) levels in THP-1 monocytes from DM2 patients with or without DN compared to healthy controls (*p* < 0.05). Pretreatment with 1,25(OH)_2_D_3_ blocked the changes of NF-κB p65 and IκB levels induced by LPS and IL-15. 

Treatment with LPS and IL-15 also resulted in a significant increase in IL-6 and MCP-1 levels in DM2 patients with or without DN compared to healthy controls (*p* < 0.01). Pretreatment with 1,25(OH)_2_D_3_ resulted in a significantly decreased IL-6 secretion by monocytes, blocking the pro-inflammatory effects of LPS and IL-15. Based on these results, the mechanism of action is likely to be via the TLR4/NF-κB p65-signaling pathway [80].

Extracellular matrix (ECM) is a prominent hallmark of DN, which ultimately results in renal fibrosis [23,24,81]. In comparison to control mice, STZ-induced DM mice showed a decrease in nephrin and podocin expression, in contrast to a higher accumulation of fibronectin in the glomeruli, which is a potent fibrogenic factor [82]. A reduced expression of nephrin and podocin was associated with increased proteinuria and deterioration of renal function [83]. 

The increased proteinuria in DN rats is also associated with an increased expression of transient receptor potential cation channel, subfamily C, member 6 (TRPC6) [84]. In addition, VDR levels were significantly lower in diabetic mouse podocytes compared to control mice, while paricalcitol-treated mice showed a similar VDR expression as untreated control mice. VDR-KO mice developed more severe grades of albuminuria, renal histopathologic changes, an increase in fibronectin, and a decrease in nephrin compared with diabetic mice [82]. 

Human transgenic VDR was able to attenuate these changes significantly. Upregulation of VDR restores the slit components, nephrin and podocin, by inhibiting the fibronectin synthesis caused by hyperglycemia [82]. Compared to diabetic wild-type mice, more fibronectin and less nephrin were present in VDR KO mice. A more severe renal injury was also characterized by increased renin, angiotensinogen, TGF-β, and connective tissue growth factor [85]. 

Hyperglycemic conditions stimulate the expression of TGF-β in rat glomerular mesangial cells, leading to accumulation in the ECM [86]. TGF-β1 is involved in histone deacetylase 5 (HDAC5)-regulated EMT in renal tubular cells [87]. Vitamin D treatment repressed the production of TGF-β in the ECM (*p* < 0.01) and after knocking down VDR, the effect of vitamin D therapy was partially repressed (*p* < 0.01) with increasing intracellular TGF-β levels [81]. 

Treatment with 1,25(OH)_2_D_3_ inhibited high glucose-induced fibronectin production in cultured mesangial cells and increased nephrin expression in cultured podocytes [85]. Vitamin D reduces macrophage infiltration, inhibits M1 macrophage activation, while enhancing M2 macrophage phenotype to protect against podocyte injury [83]. In the early stages of DN, calcitriol could ameliorate podocyte injury by inhibiting enhanced TRPC6 expression [84]. High glucose-induced EMT in human renal proximal tubular cells showed a significant increase in collagen type I protein/mRNA (*p* < 0.01), which decreased significantly after treatment with 1,25(OH)_2_D_3_ (*p* < 0.05) [81].

Nephrin is a cell surface receptor that plays an important role in cell-cell adhesion and signaling in glomerular podocytes, which contributes to renoprotection. In ex vivo isolated rat glomeruli, nephrin has been associated with the p85α regulatory subunit of nephrin-phosphatidylinositol 3-kinase (PI3K). PI3K or lipid kinase produces PIP3, which is a regulator of the Akt’s translocation to the plasma membrane as a second messenger. Akt gets activated by phosphorylation modification of threonine 308 and serine 473 [88]. 

Although PI3K is definitely involved in the development of DN, the exact function of PI3K in the diabetic kidney has not yet been fully understood. Different PI3K isoforms might explain the contrasting intracellular actions [89]. In one sense, the activated PI3K/Akt pathway in renal tubular cells might regulate the epithelial to mesenchymal cell transition (EMT), cell growth, and lipid metabolism under the diabetic condition [90,91]. 

Inhibition of the PI3K/Akt pathway ameliorates the effects of high glucose on renal tubular cells through downregulation of HDAC5 [87]. On the other hand, the PI3K/Akt signaling pathway might be involved in protecting glomerular podocytes and might ameliorate proteinuria. Upon activation of the VDR, nephrin co-localized with p85α and Akt phosphorylation increased, suggesting the PI3K/Akt signaling pathway may be involved in the reversal of DN changes [17,21].

Podocalyxin (PODXL) is essential for normal podocyte structure and function. In DN rats, a reduction in the expression of PODXL in the glomeruli has been seen. After activation of VDR, there was a markable recovery of PODXL levels (*p* < 0.05). Similarly, nephrin and PODXL were significantly downregulated in high glucose-treated human glomerular epithelial cells compared to controls (*p* < 0.05), which has been linked to loss of the permselective renal barrier and proteinuria [17]. Vitamin D treatment enhanced the expression of nephrin and PODXL (*p* < 0.005). After knocking down VDR, the effect of calcitriol was inhibited for 100% and paricalcitol for 60% (see Table 1) [17]. 

One of the most important signaling pathways involved in EMT is the Wnt/β-catenin pathway [93]. The Wnt signaling pathway is a key player in regulating differentiation of cellular morphology and function, and β-catenin is a multifunctional protein involved in classic Wnt signaling [94]. Glycogen synthase kinase-3β (GSK-3β) is one of the essential regulators of the β-catenin pathway [95]. 

High glucose conditions induce podocyte injury via the prorenin receptor (PRR)-Wnt-β-catenin-snail signaling pathway [82]. A paricalcitol treatment ameliorated proteinuria in DN, induced by a physical interaction between the VDR and β-catenin in podocytes. This process inhibits β-catenin nuclear translocation and regulates target gene transcription. In addition, renal expression of Snail, a downstream effector of Wnt/β-catenin signaling, is inhibited [96].

An effect of vitamin D on the immunohistological changes by macrophage switching via the VDR-peroxisome proliferator-activated receptor γ (PPARγ) signaling pathway has been shown. In hyperglycemic conditions, RAW264.7 macrophages exhibited a significant switch from M2 to M1 phenotypes (*p* < 0.05) [97]. When macrophages were exposed to 1,25-(OH)2D3, hyperglycemia attenuated. 

Treatment with 1,25(OH)_2_D_3_ resulted in a significant upregulation of M2 markers and downregulation of M1 markers (*p* < 0.05). VDR and PPAR_γ_ mRNA and protein levels increased significantly in a vitamin D dose-dependent manner starting compared to control and hyperglycemic conditions (*p* < 0.05). After the antagonization of PPAR_γ_, the effect of vitamin D on the switch of macrophage phenotype from M2 to M1 was significantly attenuated (*p* < 0.05). 

When knocking down VDR, there was a significant elimination of the 1,25-(OH)_2_D_3_ effect on the macrophage phenotype switch (*p* < 0.05) [81]. An in vivo study with DN rats showed a significant increase in CD68-positive macrophages, triggering receptor expressed on myeloid cells 1 (TREM-1), and p-STAT1 expression in the kidney tissue (*p* < 0.005) [97]. In hyperglycemic conditions, there was also a significant upregulation of inducible nitric oxide synthase (iNOS), and downregulation of the mannose receptor. These effects were normalized after treatment with 1,25(OH)_2_D_3_. 

Vitamin D analogues may also exert antifibrotic effects by influencing TGF-β/Smad pathway. Activation of TGF-β/Smad signaling contributes significantly to both glomerular and tubulointerstitial fibrosis [98]. In an animal model, 1,25(OH)_2_-vitamin D-derived synthetic ligands inhibited renal fibrosis by reducing TGF-β/SMAD signaling [99]. Vitamin D might exert this effect by recruiting the protein phosphatase 1A (PPM1A)/VDR complex to Smad3 [100].

By activating the p38MAPK signaling pathway, podocytes in DN may be structurally impaired [101]. Tubulointerstitial p-p38MAPK-positive cells increase with progressive interstitial fibrosis and inflammation in DN patients [102]. p38MAPK, which belongs to MAPK, is an intracellular transduction pathway that controls transcription of genes involved in apoptosis, differentiation, and proliferation [103]. 

Tubular injury in DN may be treated by targeting the p38MAPK signaling pathway. Experimental evidence suggests that inhibition of p38MAPK may contribute to a preserved renal function and reduced albuminuria along with a reduction in tubulointerstitial lesions, including tubular atrophy, tubular cell apoptosis and increased interstitial volume in db/db mice [104]. In DN, treatment with calcitriol increased VDR expression and blocked the activation of p38MAPK, resulting in reduced tubular epithelial apoptosis. Future research should focus on the intrinsic mechanism by which VDR regulates p38MAPK [105]. 

Oxidative stress, characterized by the production of reactive oxygen species (ROS), is another crucial element in the pathophysiology of DN. ROS can stimulate the protein kinase B (Akt)/uncoupling protein 2 (UCP2) signaling pathway [5] and the JAK/STAT signaling cascade [86], which induce excessive proliferation and the growth of glomerular mesangial cells as well as matrix proteins expression, contributing to DN. A high glucose experiment performed in a human tubular epithelium cell line showed a significant decrease in superoxide dismutase (SOD) and an increase in malondialdehyde (MDA), which are markers of oxidative stress (*p* < 0.01). 

Treatment with vitamin D (1 × 10^−7^ mmol/L 1,25(OH)_2_D_3_) in the high glucose group (30 mmol/L) resulted in a significant increase in SOD and a decrease in MDA (both *p* < 0.01) compared to the high glucose group without vitamin D supplementation. These results were significantly reversed (*p* < 0.01) after knocking down the *VDR* gene. Secondly, the effect of vitamin D on the mitochondrial membrane potential and apoptotic events was investigated. 

A decrease in mitochondrial membrane potential indicates early apoptosis of the cells. In hyperglycemic conditions, there was a significant decrease in mitochondrial membrane potential (*p* < 0.01), which was reversed after vitamin D treatment (*p* < 0.01) or after knocking down the *VDR* gene (*p* < 0.01). The renoprotective effect of vitamin D therapy has also been studied via oxidative stress response pathways, such as the protein kinase B (Akt)/uncoupling protein 2 (UCP2) signaling pathway [5]. An important regulator of cell growth and proliferation is Akt, which is known to respond to reactive oxygen stress (ROS). 

When Akt is dysfunctional, renal tubular apoptosis increases. UCP2, a mitochondrial carrier, regulates oxidative stress, mitochondrial membrane potential, and energetic cell processes. The protein levels of VDR and p-Akt were significantly lower in the hyperglycemic group compared to healthy controls and the high glucose group treated with vitamin D (*p* < 0.01). In contrast, UCP2 protein levels were significantly higher in hyperglycemic people than in healthy controls (*p* < 0.01). After treatment with vitamin D, there was a significant increase in VDR and p-Akt, and a decrease in UCP2 (*p* < 0.01). This effect was significantly reversed after knocking down the *VDR* gene. 

The protection of the kidney against ROS injury by 1,25(OH)_2_D_3_ therapy was also evaluated in renal glomerular mesangial cells of diabetic rats [86]. A high glucose environment caused a significant increase in ROS levels in glomerular mesangial cells (*p* < 0.001). The effect was partially reversed by treatment with vitamin D (*p* < 0.01). When knocking down the *VDR* gene, the vitamin D repression effect was partially inhibited (*p* < 0.01). 

In addition, in hyperglycemic conditions, there was a significant increase in p-JAK2, which reflects hyperglycemia-induced ROS. This effect was also decreased by vitamin D treatment. The same results were obtained for p-STAT1 and p-STAT3 levels. All these findings suggest that vitamin D treatment could exert its renoprotective role via a reduction in oxidative stress. 

Another possible mechanism is the inhibition of mesangial cell proliferation via the mammalian target of rapamycin (mTOR) pathway induced by DNA-damage-inducible transcript 4 (DDIT4) and tuberous sclerosis complex 2 (TSC2) protein [15]. Diabetes increases mTOR activity in the kidney, probably due to reduced AMP-activated protein kinase signaling [100] or decreased interaction between glyceraldehyde 3-phosphate dehydrogenase and Rheb [106], each of which promotes mTOR activation by Rheb [107]. 

The resultant increase in mTOR activity leads to enhanced cellular metabolism and growth and contributes to enhanced ECM expansion [100]. mRNA and protein expression levels of DDIT4 were reduced in STZ-induced diabetic rats with DN compared to healthy controls. After treatment with 25(OH)D_3_, there was an increase in DDIT4 expression levels. In DN rats, there was also downregulation of TSC2 protein levels, a negative regulator of the mTOR pathway, which improved after treatment with 1,25(OH)_2_D_3_ (*p* < 0.05)_._ On the other hand, the opposite pattern could be seen for p-Akt, which is a positive regulator of the mTOR pathway (*p* < 0.05) [10]. 

Vitamin D might also exert renoprotective effects by affecting the RhoA/Rho associated protein kinase (ROCK) pathway, which was investigated in an in vitro study with human renal proximal tubular cells [92]. Hyperglycemic conditions induced a significant RhoA/ROCK activation in a dose-dependent manner (*p* < 0.01). After treatment with 1,25(OH)_2_D_3_, there was a marked attenuation of RhoA mRNA and protein levels (both *p* < 0.05), as well as of ROCK activity compared to hyperglycemic conditions (*p* < 0.01).

## 5. The Genetics behind the Potential Renoprotective Effect of Vitamin D in Diabetic Nephropathy

Next to the molecular mechanisms behind the development of DN, several studies investigated possible associated genetic underlying mechanisms. Investigating the role of the *mTOR* gene in the onset of DN [23], a significantly upregulated expression of the *mTOR* gene in DN rats was observed compared to diabetic controls (*p* < 0.05). Treatment with vitamin D, insulin, and the combination showed significant downregulation of the *mTOR* genes in diabetic rats (*p* < 0.05) but remained significantly higher than in the control group (*p* < 0.05). 

A role for the glutamine-fructose-6-phosphate transaminase 1 (*GFAT*) gene in the renoprotective effects of vitamin D has been found [108]. The expression of the *GFAT* gene was significantly lower in diabetic rats treated with 20,000 IU/kg of vitamin D compared to healthy controls and diabetic rats without treatment (both *p* < 0.05). The gene expression of aldose reductase (*AR*) and advanced glycation end product (AGE) cellular receptor (*RAGE*) were not significantly different in this study.

One of the genes closely associated with VDR is the protein tyrosine phosphatase non-receptor type 2 (*PTPN2*) gene. This gene is responsible for the expression of PTPN2, also known as T cell protein tyrosine phosphatase (TCPTP), which is an anti-inflammatory cytokine. In vitro analysis with human acute monocytic leukemia cells (THP-1) showed that a high glucose environment evoked a significant inflammatory response (MCP-1, IL-6, and TNF-α in combination with decreased PTPN2) (*p* < 0.05 and *p* < 0.001, respectively), VDR expression did not change significantly. 

In serum and peripheral blood mononuclear cells (PBMCs) isolated from T2DM patients, the expression of PTPN2 correlated significantly but negative with UACR (β = −0.398, *p* < 0.001) and was significantly positive with VDR mRNA (β = 0.577, *p* = 0.022) and 25(OH)D_3_ (β = 0.185, *p* < 0.001) [70]. Both VDR and PTPN2 mRNA levels were significantly lower in normoalbuminuric, microalbuminuric, and macroalbuminuric diabetics compared with healthy controls. 

There was a significant upregulation of both VDR and PTPN2 expression in THP-1 cells treated with paricalcitol (0.2 ng/mL for 6 h) as well as a reduction of inflammatory cytokines (*p* < 0.05). After knocking down the *PTPN2* gene, there was a further significant increase in inflammatory cytokines (*p* < 0.05). Even treatment with paricalcitol failed to exert anti-inflammatory effects after knocking down the *PTPN2* gene. 

To further explain the effect of hyperglycemia on JAK/STAT signaling [86], the influence on downstream genes (suppressor of cytokine signaling 1 (*SOCS1*), *SOCS3*, and type IV collagen) was explored in rat glomerular mesangial cells. All three genes were upregulated in hyperglycemic conditions (*p* < 0.01), and the expression was repressed after vitamin D therapy (*p* < 0.01). The relationships between four single nucleotide polymorphisms (SNPs) (Bsml (rs1544410), ApaI (rs7975232), TaqI (rs731236) and FokI) in the *VDR* gene and DN have recently been investigated [9,92,109].

BsmI polymorphisms appear to be associated with DM2 and DN in the Chinese population. In patients with DM2, the BB + Bb genotype and B-allele frequency were significantly higher compared with controls (*p* = 0.008; *p* = 0.015, respectively). Even more, the BB + Bb genotype and the B-allele frequency were significantly higher in the early onset DN compared with the late-onset DN (*p* = 0.007; *p* = 0.016, respectively). This might suggest that the BsmI phenotype could be a risk factor for early-onset DN (BB + Bb phenotype, OR: 2.394; 95% CI: 1.032–5.333) [109]. 

However, in a Tunesian case-control study with 100 patients with chronic renal failure in ESRD and 149 healthy subjects, no association was found between the BsmI *VDR* gene polymorphism and the development of renal nephropathy [8]. The F-allele in the FokI polymorphism was associated with a 1.8-times higher risk of DN in Chinese patients (OR: 1.825, 95% CI: 1.259–2.646, *p* < 0.002) [109]. The study of Liu et al. [10] showed a significant association between FokI polymorphism of the *VDR* gene and the risk for DN1. The underlying biological mechanism of this process remains unclear. 

The f allele represents the restriction site of the FokI gene. When the f allele is absent, the gene is transcribed into a shortened length resulting in dysfunction of the VDR gene. However, due to the complex interaction between genes and environmental factors and other genes, the result remains undetermined [10]. In the Tunisian population, the TT genotype of the FokI polymorphism was associated with a lower risk of renal nephropathy development [8]. 

The ApaI polymorphism showed no significant correlation with DM2 or DN [109]. The effect of several combinations of *VDR* genotype polymorphisms showed that the BBFFAATt combination of VDR polymorphisms was significantly more frequent in the DN group compared to healthy controls (*p* = 0.046; OR: 0.936; 95% CI: 0.890–0.983). The BbFFAaTt polymorphism was significantly more frequent in the DN group compared to DM2 patients (*p* = 0.018; OR: 2.575; 95% CI: 1.119–5.923) [9].

## 6. Conclusions

Vitamin D deficiency has been related to DM and its complications [54]. As vitamin D levels are significantly lower in DKD patients compared with diabetic patients without DKD and CKD patients without DKD, there seems to be a close association between vitamin D deficiency and DN [54]. The remaining question is whether vitamin D deficiency is a cause of DKD or merely a consequence. Patients with CKD produce less 1,25(OH)_2_D_3_ due to impaired 1α-hydroxylase activity [110,111]. In patients with T1DM or T2DM, vitamin D deficiency increases the risk of developing DKD [28,35,40,44,46,47,48,49,50,51], possibly due to the direct cellular effects, leading to podocyte loss and glomerulosclerosis [53]. 

Complementary, vitamin D deficiency is independent of the degree of albuminuria and is not a consequence of renal deterioration in DKD patients [112]. In contrast to this finding, no significant correlation between vitamin D levels and UACR has been reported by other research groups, which might be explained by the small number of study patients in these trials [53,56]. Several well-designed observational and interventional studies have shown a recovery of renal function after vitamin D therapy [27,56,62,63,64,65], which was not confirmed by others [25,53,66,67,68]. 

Despite the fact that vitamin D shows a crucial role in the development of DKD in multiple animal and human studies, the possible renoprotective role of vitamin D in the process of developing DKD and reversal of already existing renal damage remains uncertain. Several interventional studies have published controversial results regarding this renoprotective effect. A potential underlying reason for these controversial results is the analytical variability and the lack of standardization of 25(OH)D assays. 

The lack of assay standardization is the major source of bias, thus, making it impossible to pool research results to develop consensus cut-off points [113]. The Vitamin D Standardization Program (VDSP) was established in 2010 to overcome these problems. There are currently three Joint Committee for Traceability in Laboratory Medicine (JCTLM)-recognized reference measurement procedures (RMPs) for the determination of 25(OH)D_2_ and 25(OH)D_3_ based on isotope dilution-liquid chromatography-tandem mass spectrometry (ID-LC-MS/MS), making it possible to standardize 25(OH)D measurements. 

The introduction of an international Vitamin D standardization certification program (VDSCP) has led to an impressive improvement in the number of standardized 25(OH)D assays [114]. However, even today, several immunoassays suffer from analytical issues leading to continuing problems with the quality of 25(OH)D measurements, e.g., different affinities for 25(OH)D_2_ and 25(OH)D_3_, cross-reactivity with 24,25(OH)_2_D, and matrix and/or patient-dependent biological variations [14].

There are currently no recommendations about the optimal dosage or timing of vitamin D analogues in DN. There is a call for more large-scale, long-term, randomized, and controlled human studies evaluating the influence of vitamin D and its analogues on the development of DKD, the delay in the decline of eGFR, the progression to ESRD, and mortality. 

It would be interesting to investigate the role of vitamin D in the prevention of DN in diabetic patients with a high risk of development of microalbuminuria, based on a urinary proteomic risk classifier (CKD273) score [115]. Further study is needed to determine the role of VDR activators on the clinical symptoms and consequences of DN. Awaiting the results of ongoing clinical trials, we hypothesize that supplementation with vitamin D could be initiated in DM patients with vitamin D deficiency to prevent or potentially revert the progression of DN. 

In agreement with the National Kidney Foundation-Kidney Disease Outcomes Quality Initiative (NKF-KDOQI) publications concerning bone metabolism and disease in patients with CKD, vitamin D regimens might consist of supplementation with ergocalciferol, 50,000 IU, for four weekly doses, and then monthly for 5 months to reach 25(OH)D levels of ≥ 30 ng/mL [116]. There are a few hypotheses concerning the underlying molecular mechanisms (inflammation, oxidative stress, and ECM accumulation) in the pathophysiology of DN, but the evidence remains limited. 

Regarding the genetics behind DN, multiple polymorphisms (BsmI, ApaI, and FokI) have recently been investigated, although the exact impact on DKD development has not yet been determined. Future studies should focus on genetic factors influencing vitamin D concentrations in DM patients with CKD. Research into the interindividual variability in the metabolism of vitamin D and different responses to supplementation, which might result from genetic polymorphisms, could be of interest as well [117].

## Figures and Tables

**Table 1:** Overview of possible molecular mechanisms of development of diabetic nephropathy in animals. HC: hyperglycemic conditions; VDR-KO: vitamin D receptor gene knock down; PODXL: podocalyxin; TGF-ß: transforming growth factor-ß; * Compared to HC; and ** Compared to HC + vitamin D.

Molecular Marker	HC	HC + VDR Activation *	HC + Vitamin D + VDR-KO **	Reference
Nephrin	↓	↑	/	[17]
	↓	↑	↓	[22]
PODXL	↑	↓	↑	[17]
Fibronectin	↑	↓	↑	[22]
	↑	↓	↑	[86]
	↑	↓	/	[92]
TGF-ß	↑	↓	↑	[86]
Podocin	↓	↑	/	[22]
Collagen type I	↑	↓	/	[92]

## Data Availability

Not applicable.
